# Novel mutation in carnitine palmitoyltransferase 1A detected through newborn screening for a presymptomatic case in China: a case report

**DOI:** 10.1186/s13052-021-01094-5

**Published:** 2021-07-07

**Authors:** Yi Gan, Fei Yu, Haining Fang

**Affiliations:** 1 Pediatric Department, Maternal and Child Health Hospital of Hubei Province, Tongji Medical College, Huazhong University of Science and Technology, Wuhan, People’s Republic of China; 2 Neonatal Genetic Metabolic Disease Screening and Treatment Center in Hubei Province, Wuhan, People’s Republic of China

**Keywords:** Carnitine palmitoyltransferase 1A deficiency, Tandem mass spectrometry, Gene mutation, Newborn screen, Case report

## Abstract

**Background:**

Carnitine palmitoyltransferase 1A (CPT1A) deficiency is a rare mitochondrial fatty acid oxidation (FAO) disorder that results in hypoketotic hypoglycemia and hepatic encephalopathy. It is caused by mutation in *CPT1A*. To date, only two symptomatic cases of CPT1A deficiency have been reported in China.

**Case presentation:**

A newborn male, without any disease-related clinical manifestations, was diagnosed with CPT1A deficiency through newborn screening. Increased free carnitine levels and a significantly increased C0/(C16 + C18) ratio were detected by tandem mass spectrometry, and subsequently, mutations in CPT1A were found by gene sequence analysis. The patient was advised a low-fat, high-protein diet and followed up regularly. During three-years of follow-up since, the patient showed normal growth velocity and developmental milestones. Whole-exome sequence identified two mutations, c.2201 T > C (p.F734S) and c.1318G > A (p.A440T), in the patient. The c.2201 T > C mutation, which has been reported previously, was inherited from his father, while the c.1318G > A, a novel mutation, was inherited from his mother. The amino acid residues encoded by original sequences are highly conserved across different species. These mutations slightly altered the three-dimensional structure of the protein, as analyzed by molecular modeling, suggesting that they may be pathogenic.

**Conclusion:**

This is the first case of CPT1A deficiency detected through newborn screening based on diagnostic levels of free carnitine, in China. Three years follow-up suggested that early diagnosis and diet management may improve the prognosis in CPT1A patient. In addition, we identified a novel mutation c.1318G > A in *CPT1A,*and a possible unique to Chinese lineage mutation c.2201 T > C. Our findings have expanded the gene spectrum of this rare condition and provided a basis for family genetic counseling and prenatal diagnosis.

## Background

**Fig. 1:**
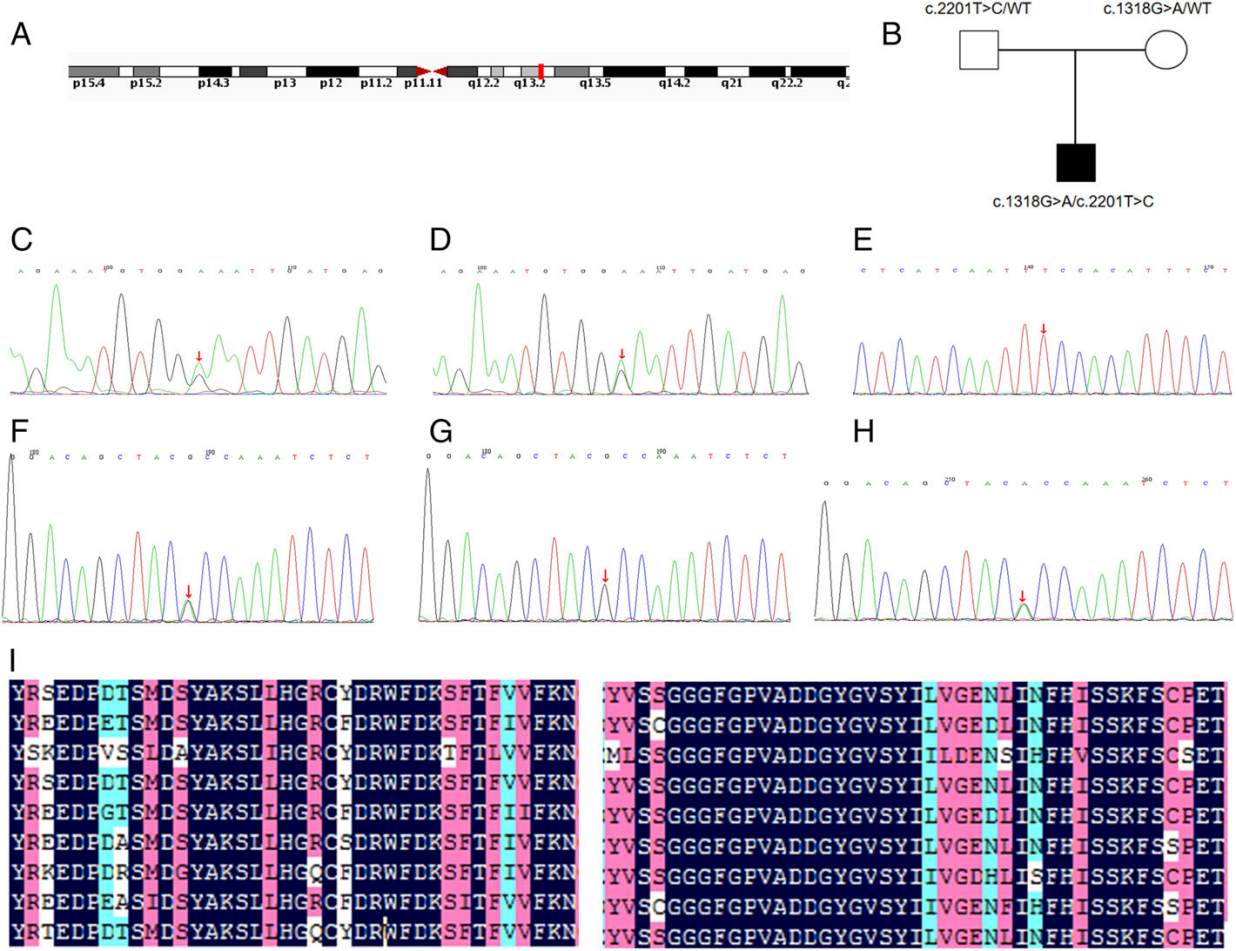
**(A)** Pedigree chart of the family. **(B)** CPT1A Gene in genomic location:11q13.3 **(C-H)** Consequence of DNA analysis: **(C)** The patient carrying 2201 T > C mutation. **(D)** His father carrying 2201 T > C mutation. **(E)** His mother: normal. **(F)** The patient carrying 1318 T > C mutation. **(G)** His father: normal. **(H)** His mother carrying 1318 T > C mutation. **(I)** Multiple species alignment analysis showed the high evolutionary conservation of amino acid sequence at the mutation site

Carnitine palmitoyltransferase 1A (CPT1A, EC# 2.3.1.21) deficiency is a rare autosomal recessive inherited disorder of the carnitine cycle (MIM #255120) [1]. It is caused by mutations in the gene coding CPT1A, which is located in chromosome 11q13.3 (Fig. 1A) [2]. This enzyme is essential for transport of long-chain fatty acyl-CoA esters into the mitochondria for subsequent beta-oxidation. Loss of CPT-1A activity diminishes the intra-mitochondrial substrate levels for fatty acid beta-oxidation, thereby impairing energy generation [1]. Therefore, patients with CPT1A deficiency usually present hypoketotic hypoglycemia and hepatic encephalopathy after long periods of fasting. As reported, the onset usually occurs within 18 months by birth, following various symptoms including hypoketotic hypoglycemia, lethargy and seizures [3]. In this report, we present the first presymptomatic case of CPT1A deficiency detected through newborn screening in China and a novel mutation has been found. Moreover, we identified a novel mutation associated with this disorder.

## Case presentation

### Clinical history

A male child was born of a normal pregnancy and natural delivery in our hospital and is the only child of his parents. His gestational age was 39 weeks, Apgar score 10/1, 10/5, 10/10, birth weight 3500 g. Plantar blood (three droplets) was collected from the child was dropped onto the filter paper (Whatman S&S903) for newborn screening test including tandem mass spectrometry (MS/MS) on the 3rd day of birth. When the boy was 30 days old, he was recalled for retesting on the phone call because of abnormal result (No specific report). When the boy was 44 days old, the results came out showed increased blood free carnitine(C0) level of 128.1 mmol/L (ref < 50 mmol/L) and increased C0/(C16 + C18) ratio of 512.4 (ref < 42). These abnormal results were confirmed by testing again on day 51 after birth, the results showed 65.86 mmol/L free carnitine (ref < 100 mmol/L) and a significantly increased C0/(C16 + C18) ratio of 1423.97 (ref < 100), which were consistent with CPT1A deficiency [3]. Based on these results, the boy was thoroughly examined. Cranial MRI showed no significant abnormalities echocardiogram was normal. The urine organic acid was checked for two times without any abnormality. Laboratory findings for blood sample were: pH,7.4; base excess, 4.9 mmol/L (ref 4.0–2.0 mmol/L); bicarbonate, 18.4 mmol/L (ref 21.8–26.2 mmol/L); ammonium, 29.2 mmol/L (ref < 47 mmol/L); lactate, 2.9 mmol/L (ref 0.5–2.2 mmol/L); thrombocytes 333 × 10^9^/L (ref 160–360 10^9^/L); hemoglobin, 111 g/L (ref 110–160 g/L); alkaline phosphatase, 306 IU/L (ref 55–425 IU/L); alanine aminotransferase 38.7 IU/L (ref 5–50 IU/L); bilirubin, 52.2 μmol/L (ref 0–24 μmol/L);creatin kinase,82 U/L(ref 50–310 U/L); creatine kinase isoenzyme MB, 30.5 U/L(ref 0–25 U/L); plasma glucose, 4.6 mmol/L (ref 3.3–5.5 mmol/L). These blood tests were repeated after 3 days and the results were normalized after treatment. The patient was discharged when acylcarnitine became normal. The diagnosis of CPT1A deficiency was considered and confirmed by gene sequence (MyGenostics,Beijing, China).

The patient was advised a low-fat, high-protein diet (no accurate proportion) and followed-up regularly. Foods full of medium chain fatty acids including deeply hydrolyzed formula and palm oil were recommend. On earlier occasions when he fell sick, hypoglycemia was prevented by early intervention with glucose infusion. Every 3 months, the patient was examined by a specialist to evaluate if he suffered any neurological damage due to possible episodes of hypoketotic hypoglycemia that is associated with CPT1A deficiency. There were no motor retardation and hypotonia. At 12 months of age, the boy could walk and talk. During the 3 years of follow-up since, his psychomotor development has been appropriate for his age.

### Molecular genetic findings

**Fig. 2:**
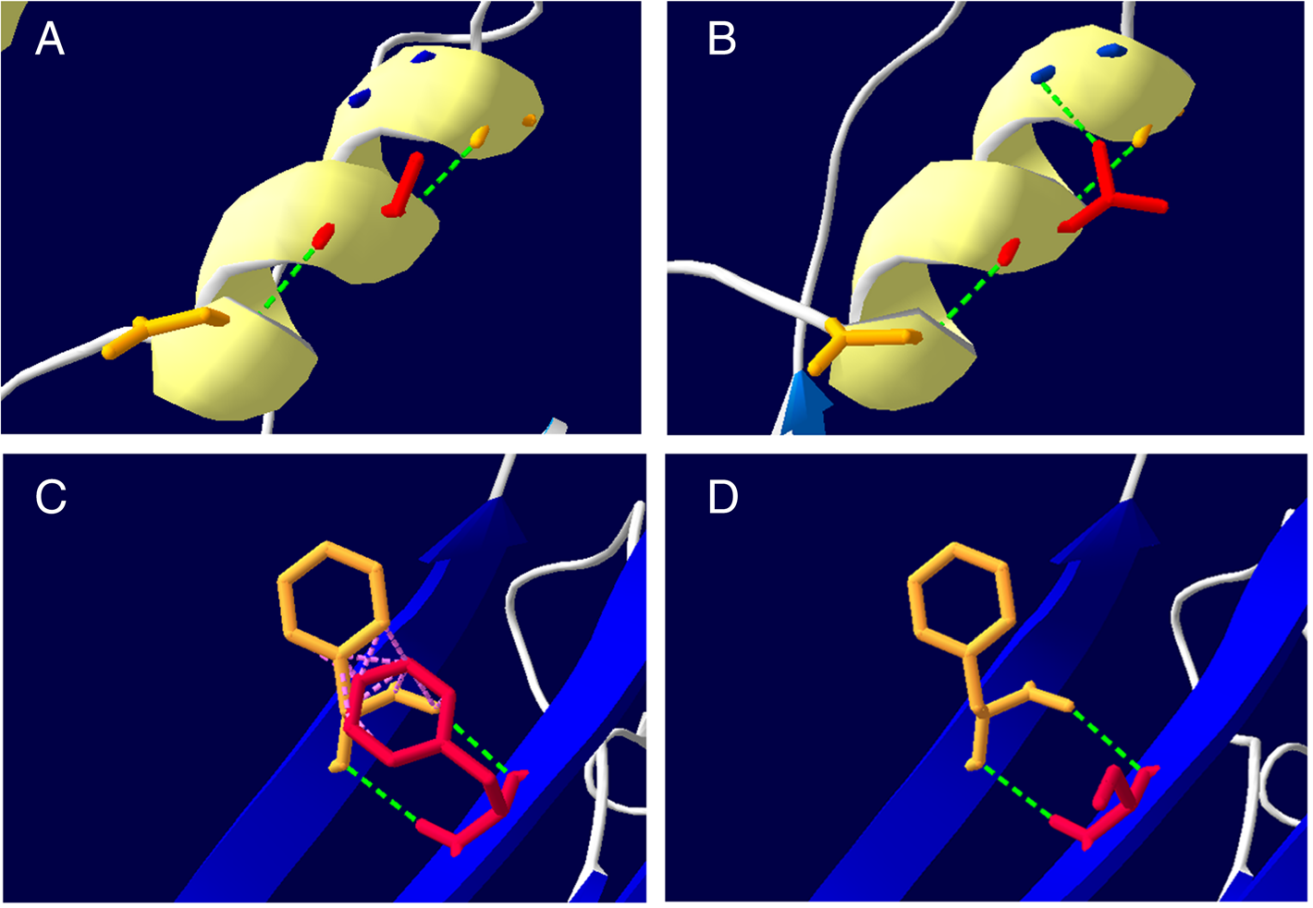
3-D structure of the wild type and p.A440T/p.F734S CPT1A proteins with prediction software. **(A)** Before p.A440T mutation. **(B)** After p.A440T mutation. **(C)** Before p.F734S mutation. **(D)** After p.F734S mutation. (The green dotted lines are hydrogen bond, and the red dotted lines are hydrophobic bond)

*CPT1A* (Ensemble gene: ENST00000110090) was sequenced for the patient after obtaining written informed consent of his parents. The results showed two mutations: c.2201 T > C (p.F734S) and c.1318G > A (p.A440T) in exons 18 and 11, respectively. Then family screening of these mutations for patient’s parents was performed. Results showed that the c.2201 T > C mutation was transmitted from his father, while c.1318G > A mutation was transmitted from his mother (Fig. 1B, C-H). These variations were not listed in the SNP database (http://www.ncbi.nlm.nih.gov/projects/SNP/), Human Gene Mutation Database Professional (http://www.hgmd.cf.ac.uk/ac/index.php). However, c.2201 T > C has been reported once in one case of CPT1A deficiency in China [4], while c.1318G > A has not been previously reported. According to PolyPhen2 (http://genetics.bwh.harvard.edu/pph2/), the c.2201 T > C mutation was predicted to be “probably damaging” (score, 0.995) and c.1318G > A was predicted to be “possibly damaging” (score, 0.875), suggesting that both mutations may cause disease. The sequence data revealed that both the mutations detected in our patient were missense mutations causing p.F734S and p.A440T replacement. Further, we conducted molecular modeling to predict the effect of these mutations on the protein structure of CPT1A (Fig. 2). We found that replacement of Thr440 with Ala440 results in an additional hydrogen bond between Thr440-MET436, and replacement of Ser734 with Phe734 results in the loss of hydrophobic bond force between Ser734-Phe549. As hydrogen and hydrophobic bond play an important role in maintaining protein spatial conformation and stability, these subtle changes in spatial structure may affect protein function. These data suggest that the mutation causing these substitutions may not be polymorphisms, but disease-causing mutations.

## Discussion

CPT1A deficiency is a rare metabolic disease that affects fatty acid oxidation (FAO), and in the majority of cases, patients are diagnosed only after the appearance of clinic symptoms. Most patients present these symptoms by the age of 2 years with hypoketotic hypoglycemia induced by fasting or illness [5]. This is usually accompanied by liver dysfunction; transient lipemia and renal tubular acidosis may also be present [1]. As our patient underwent the newborn screening at the age of 3 days, indicators of CPT1A deficiency, namely, increased free carnitine and a significantly increased C0/(C16 + C18) ratio, were detected early. The diagnosis of CPT1A deficiency was confirmed by gene sequence analysis. Dietetic management and avoidance of prolonged fasting were recommended to improve the patient’s clinical outcome [5]. Therefore, our patient developed normally, without severe metabolic crisis, till date. Indicate that early recognition of this disease is crucial for prognosis.

CPT1A deficiency is caused by mutation in the gene encoding *CPT1A.* Analysis of *CPT1A* is necessary for accurate diagnosis. So far, more than 30 mutations in *CPT1A*, responsible for the CPT1A deficiency, have been identified [6]. Our patient carried two missense mutations c.2201 T > C (p.F734S), previously reported in one Chinese patient [4], and c.1318G > A (p.A440T), a novel mutation. The amino acid residue at 734 and 440 in CPT1A is highly conserved in bovine, chicken, chimpanzee, goat, horse, pig, rat, and macaque (Fig. 1I), suggesting that these loci play key roles in CPT1A normal function. According to prediction software analysis these mutations do not appear to be polymorphisms, but are more likely to be disease-causing mutations.

As we have listed the CPT1A mutations reported so far, based on the geographic region in Table 1. Most mutations seem to be unique or restricted to only a few pedigrees, except c.2129G > A and c.1436C>T [2]. While c.2129G > A (p.G710E), a homozygous mutation associated with disease severity, is mainly found in Alaskan and Hutterite populations in the USA [19]. c.1436C > T mutation (p.P479L) was more common in northern Canada, Greenland, Colombia, as well as the native Alaskan population [20]. Most affected individuals in these populations are homozygous for this variant [20]. p.P479L pathogenic variant is generally regarded as non-pathogenic with relatively high residual enzymatic activity but still associated with infant mortality [18, 21]. Exon and multi exon deletions are rarely reported [17]. The incidence of this disorder appears to be quite low in other regions. The true meaning of this variation in regions remains unknown. To date, only two cases of CPT1A deficiency have been reported in China [4, 8], and both were diagnosed after the patients (> 1 year old) exhibited symptoms of hypoglycemia followed by diarrhea and fever. Moreover, there is almost complete genetic heterogeneity of disease-causing *CPT1A* variations with each affected family demonstrating novel variation(s) of *CPT1A* [1]. Therefore, analysis of the entire *CPT1A* is required to confirm an abnormal newborn screen and the disease-causing nature of the abnormal genotypes needs to be carefully interpreted [7, 9]. As p.F734S mutation has been reported once in a Chinese patient with heterozygous gene mutation, it may be a unique to Chinese lineage.

**Table 1:** Reported CPT1A Pathogenic Variants

Country	Variants	case	reference
Alaska	c.1436C > T;	^a^	[7]
China	c.281 + 1G > A/IVS2_IVS5del; c.1787 T > C/c.2201 T > C	2	[4, 8]
Korean	c.837_838ins(T)/c.947G > A	1	[5]
Denmark	c.167C > T	1	[9]
Finland	c.1364A > C; c.1364A > C/c.1493A > C; c.1463C > T	6	[10]
Japan	c.1339C > T/c.2156G > A; c.96 T > G/c.1079A > G; 2027–2028 + 2del; c.1425G > A/c.1494 T > G	4	[6, 11, 12]
Netherlands	c.1737C > A; c.478C > T; c.1600delC; c.1361A > G	4	[13, 14]
American	c.1393G > T; c.1027 T > C; c.478C > T; c.946C > G/?; c.986C > T; c.1163 + 1G > A; c.823G > A/c.912C > G; c.367C > T; c.2129G > A^#^	8 + ^b^	[1, 15, 16]
France	c.298C > T; C.1241C > T/1493A > G; IVS14 + 3 kb; c.1876-1G > A	4	[17]
Indian	c.1069C > T/c.1451 T > C	1	[18]

Those variants followed the standard naming conventions of the Human Genome Variation Society (http://varnomen.hgvs.org/)

^a^Inuit mutation

^b^Hutterite mutation

Different mutations lead to different structural and functional changes. Missense mutations p.A275T, p.A414V, and p.Y498C of *CPT1A* were located away from the active site and mostly affected the stability of the protein itself and/or of the enzyme–substrate complex which exhibited decreased catalytic efficiency. In contrast, mutants p.G709E and p.G710E affect glycine residues, which are critical to the structure of the hydrophobic core of the catalytic site and the chain length specificity of the CPT isoform, which greatly impair the catalytic function of CPT1A, without affecting enzyme localization and stability [22]. Mutations like p.R357W, p.L484P, and p.C304W are associated with decreased protein stability. p.C304W, p.P479L, and p.delR395 were found to inactivate CPT1A [18]. The p.P479L variant affects the malonoyl-CoA binding site of the enzyme, resulting in a 25% ~ 50% decrease in CPT1A activity. Another study found that mutant p.E360G caused a decrease in CPT1A protein levels [11]. Mutations in conserved arginines and tryptophans of CPT1A have been reported to reduce enzyme activity, such as Arg388, Arg451 Trp391 and Trp452. Overall, all CPT1A mutations that lead to loss of function or stability are present in the C-terminal catalytic domain (123–773 aa) [23]. Our patient were missense mutations causing p.F734S and p.A440T replacement. Using 3-dimensional modeling it was demonstrated that the mutation p.F734S and p.A440T may change the spatial structure which may affect protein function.

Newborn screening programs, which allow early detection of metabolic markers in dried whole blood spots when the newborn is catabolic, are therefore very important [3]. Newborn screening for CPT1A deficiency is now mandatory in 32 states of the USA and available in 16 more states and 5 Canadian Provinces [24]. In Italy, since 2016, extended neonatal screening has been mandatory throughout the country for about 40 inherited metabolic diseases [25]. However, screening for CPT1A deficiency is not included in the newborn screening program in every province in China. The routine neonatal metabolic screening programs include phenylketonuria, congenital hypothyroidism, hereditary glucose-6-phosphate dehydrogenase deficiency, congenital adrenal hyperplasia, thalassemia, and neonatal hearing screening. Since 2016, Central China (Hubei Province) newborn screening program has included screening for disorders of fatty acid oxidation using MS/MS, and more than 120,000 newborn children have been tested so far. The MS/MS scanning(API3200MD,AB, America) method we used was mutiple reaction monitoring(MRM). However, only five large neonatal screening centers have been set up so far in Hubei Province, and the feedback mechanism are not perfect in some regions, Which may lead to prolonged screening time and delay in diagnosis. If abnormal results occur, the child will be referred to a pediatric endocrinologist for further examination.

CPT1A deficiency is characterized by significant decrease in the synthesis of all acyl carnitine species, as well as increased levels of free CO and C0/(C16 + C18) on newborn screen blood spots. The MS/MS is used to detect elevated free carnitine to C16 + C18 ratio, which is characteristic of CPT1A patient [3]. Urine organic acids show elevated levels of dodecanodi acid in acute crisis and subsequent days [13]. In this case, the results of urine organic acid were normal, possibly the patient was not in the acute stage. In this case, plantar blood was collected 3 days after birth, and abnormalities came out at 30 days old, which is a long and dangerous time. The time of newborn screening test in our province should be improved.

According to the data from newborn screening programs in Australia, Germany, and the USA the incidence of CPT1A deficiency may be as low as 1:750,000 to 1:2,000,000 [26]. Alaska infants have a high prevalence of CPT1A c.1436C > T mutation in newborn screening program [27]. There is evidence suggests that homozygous infants with this variant have impaired fasting tolerance [28] and increased risk of infant mortality [7]. Five of 152 homozygous infants died for c. 1436 c > T mutation, two of 219 heterozygous infants died, and zero of 245 non-carriers died. In a study of Arctic populations with whole-genome high-coverage sequence, the CPT1A variant was identified as deleterious and associated with increased infant mortality in surrounding Arctic populations [21]. There are only two cases without death in China so far. The present case is the first presymptomatic CPT1A deficiency case detected through newborn screening in China. As neonatal MS/MS screening becomes more comprehensive across the country, it is possible that the detection rate will be higher.

CPT1A deficiency is a long-chain fatty acid oxidation disorder. Most individuals have a residual enzyme activity of 1–5% [23]. When energy demand increases, clinical manifestations usually occur in individuals with concurrent fever or gastrointestinal disease; Symptoms usually appear quickly. Prolonged fasting should be avoided or a sufficient level of glucose should be supplied, especially during periods of fever or gastrointestinal illness. Potentially hepatotoxic drugs such as valproate and salicylate should not be given, even though adverse pharmacological reactions have not been reported in individuals with CPT1A deficiency [2]. Previous reports has indicated that following a strict dietary regimen allows the CPT1A-deficient infant to lead a healthy life with normal growth and development [3]. Consistent with this reports, our patient was diagnosed at an early age, received timely intervention, and showed a normal growth trend. However, There may not be clinical trials for this disorder. The newborn metabolic screen is important for early diagnosis and treatment. Considering the simplicity of this method, it can be implemented across the country. Moreover, in the present cases, genetic counseling was recommended for the parents, should they wish to have another baby.

## Conclusion

In conclusion, we used neonatal screening using MS/MS to diagnose CPT1A deficiency in presymptomatic newborn. The early diagnosis and diet management improved the prognosis in our patient. So we need to expand the scope of neonatal MS/MS screening in whole country, improve the speed of the test time, and prefect the feedback mechanism in the future. Further, we identified a novel mutation c.1318G > A in *CPT1A*, which is probably disease-causing mutation, and c.2201 T > C mutation may be unique to Chinese lineage. Thus, our finding has expanded the gene spectrum of this rare condition and provided a basis for genetic counseling of the family and prenatal diagnosis.

## Data Availability

The datasets used and analyzed during the current study are available from the corresponding author on the reasonable request.

## Abbreviations

CPT1A: Carnitine palmitoyltransferase 1A
FAO: fatty acid oxidation
MS/MS: tandem mass spectrometry
MRM: mutiple reaction monitoring

## References

[1] Bennett MJ, Boriack RL, Narayan S, Rutledge SL, Raff ML (2004). Novel mutations in CPT 1A define molecular heterogeneity of hepatic carnitine palmitoyltransferase I deficiency. Mol Genet Metab.

[2] University of Washington, Seattle. Bennett MJ, Santani AB. Carnitine palmitoyltransferase1A deficiency. In: Pagon RA, Adam MP, Ardinger HH, Wallace SE, Amemiya A, Bean LJH, et al, eds. GeneReviews(R). Seattle: Seattle University of Washington, 2005. https://www.ncbi.nlm.nih.gov/books/NBK1527/

[3] Dykema DM (2012). Carnitine palmitoyltransferase-1A deficiency: a look at classic and arctic variants. Adv Neonatal Care.

[4] Dong C, Hu Y, Shen D (2017). Clinical features and genetic analysis of a case with carnitine palmitoyltransferase 1A deficiency. Chin J Med Genet.

[5] Lee BH, Kim YM, Kim JH, Kim GH, Kim JM, Kim JH, Woo KH, Yang SH, Kim CJ, Choi IH, Yoo HW (2015). Atypical manifestation of carnitine palmitoyltransferase 1A deficiency: hepatosplenomegaly and nephromegaly. J Pediatr Gastroenterol Nutr.

[6] Tsuburaya R, Sakamoto O, Arai N, Kobayashi H, Hasegawa Y, Yamaguchi S, Shigematsu Y, Takayanagi M, Ohura T, Tsuchiya S (2010). Molecular analysis of a presymptomatic case of carnitine palmitoyl transferase I (CPT I) deficiency detected by tandem mass spectrometry newborn screening in Japan. Brain Development.

[7] Gessner BD, Gillingham MB, Johnson MA, Richards CS, Lambert WE, Sesser D, Rien LC, Hermerath CA, Skeels MR, Birch S, Harding CO, Wood T, Koeller DM (2011). Prevalence and distribution of the c.1436C→T sequence variant of carnitine palmitoyltransferase 1A among Alaska native infants. J Pediatr.

[8] Hui Zhang, Yuanhong Yuan, Lian Tang, et al. Clinical Characteristics of One Case of Carnitine Palmitoyltransferase 1A Deficiency and Analysis of CPT1A Gene Mutation. Journal of Pediatric Pharmacy. 2019. (11): 23–27. (in chinese). DOI:10.13407/j.cnki.jpp.1672-108X.2019.11.007.

[9] Borch L, Lund AM, Wibrand F (2012). Normal levels of plasma free carnitine and Acylcarnitines in follow-up samples from a Presymptomatic case of carnitine Palmitoyl transferase 1 (CPT1) deficiency detected through newborn screening in Denmark. JIMD Rep.

[10] Roomets E, Polinati PP, Euro L, Eskelin PM, Paganus A, Tyni T. Carrier frequency of a common mutation of carnitine palmitoyltransferase 1A deficiency and long-term follow-up in Finland. J Pediatr. 2012. 160(3): 473–479.e1. DOI: 10.1016/j.jpeds.2011.08.032. 10.1016/j.jpeds.2011.08.032 21962599

[11] Ogawa E, Kanazawa M, Yamamoto S, Ohtsuka S, Ogawa A, Ohtake A, Takayanagi M, Kohno Y (2002). Expression analysis of two mutations in carnitine palmitoyltransferase IA deficiency. J Hum Genet.

[12] S. Yamamoto, M. Kanazawa, A. Ogawa, M. Takayangi, A.Ohtake, Y. Kohno, Molecular analysis of hepatic carnitine palmitoyltransferase I deficiency: cDNA and genomic DNA analyses of infants presenting with Reye-like illness, J. Inherit. Metab. Dis. 23 (Suppl. 1) (2000) 229 (Abstract). 10.1023/A:1017326813602.

[13] Korman SH, Waterham HR, Gutman A, Jakobs C, Wanders RJ (2005). Novel metabolic and molecular findings in hepatic carnitine palmitoyltransferase I deficiency. Mol Genet Metab.

[14] IJlst L, Mandel H, Oostheim W, Ruiter JP, Gutman A, Wanders RJ (1998). Molecular basis of hepatic carnitine palmitoyltransferase I deficiency. J Clin Invest.

[15] Stoler JM, Sabry MA, Hanley C, Hoppel CL, Shih VE (2004). Successful long-term treatment of hepatic carnitine palmitoyltransferase I deficiency and a novel mutation. J Inherit Metab Dis.

[16] Dowsett L, Lulis L, Ficicioglu C, Cuddapah S. Utility of Genetic Testing for Confirmation of Abnormal Newborn Screening in Disorders of Long-Chain Fatty Acids: A Missed Case of Carnitine Palmitoyltransferase 1A (CPT1A) Deficiency. Int J Neonatal Screen. 2017;3(2). 10.3390/ijns3020010. 10.3390/ijns3020010 PMC5523953 28748224

[17] Gobin S, Bonnefont JP, Prip-Buus C, Mugnier C, Ferrec M, Demaugre F, Saudubray JM, Rostane H, Djouadi F, Wilcox W, Cederbaum S, Haas R, Nyhan WL, Green A, Gray G, Girard J, Thuillier L (2002). Organization of the human liver carnitine palmitoyltransferase 1 gene ( CPT1A) and identification of novel mutations in hypoketotic hypoglycaemia. Hum Genet.

[18] Brown NF, Mullur RS, Subramanian I (2001). Molecular characterization of L-CPT I deficiency in six patients: insights into function of the native enzyme. J Lipid Res.

[19] Prip-Buus C, Thuillier L, Abadi N, Prasad C, Dilling L, Klasing J, Demaugre F, Greenberg CR, Haworth JC, Droin V, Kadhom N, Gobin S, Kamoun P, Girard J, Bonnefont JP (2001). Molecular and enzymatic characterization of a unique carnitine palmitoyltransferase 1A mutation in the Hutterite community. Mol Genet Metab.

[20] Park JY, Narayan SB, Bennett MJ (2006). Molecular assay for detection of the common carnitine palmitoyltransferase 1A 1436(C>T) mutation. Clin Chem Lab Med.

[21] Clemente FJ, Cardona A, Inchley CE (2014). A selective sweep of a deleterious mutation in CPT1A in Arctic populations. Amer J Hum Genet.

[22] Gobin S, Thuillier L, Jogl G, Faye A, Tong L, Chi M, Bonnefont JP, Girard J, Prip-Buus C (2003). Functional and structural basis of carnitine palmitoyltransferase 1A deficiency. J Biol Chem.

[23] Isabel R (2020). Schlaepfer, Molishree Joshi, CPT1A-mediated fat oxidation, mechanisms, and therapeutic potential. Endocrinology.

[24] Fohner AE, Garrison NA, Austin MA, Burke W. Carnitine palmitoyltransferase 1A P479L and infant death: policy implications of emerging data. Genet Med. 2017;19(8):851–857. DOI: 10.1038/gim.2016.202. 10.1038/gim.2016.202 PMC5620101 28125087

[25] la Marca G. La organización del cribado neonatal en Italia: comparación con Europa y el resto del mundo [The Newborn Screening Program in Italy: Comparison with Europe and other Countries.]. Rev Esp Salud Publica. 2021 26;95:e202101007. Spanish. PMID: 33496274. 33496274

[26] Lindner M, Hoffmann GF, Matern D (2010). Newborn screening for disorders of fatty-acid oxidation: experience and recommendations from an expert meeting. J Inherit Metab Dis.

[27] Collins SA, Sinclair G, McIntosh S, Bamforth F, Thompson R, Sobol I, Osborne G, Corriveau A, Santos M, Hanley B, Greenberg CR, Vallance H, Arbour L (2010). Carnitine palmitoyltransferase 1A (CPT1A) P479L prevalence in live newborns in Yukon, Northwest Territories, and Nunavut. Mol Genet Metab.

[28] Gillingham MB, Hirschfeld M, Lowe S, Matern D, Shoemaker J, Lambert WE, Koeller DM. Impaired fasting tolerance among Alaska native children with a common carnitine palmitoyltransferase 1A sequence variant. Mol Genet Metab. 2011;104:261–4. DOI: 10.1016/j.ymgme.2011.06.017. 10.1016/j.ymgme.2011.06.017 PMC3197793 21763168

