# Correction to: Novel mutation in carnitine palmitoyltransferase 1A detected through newborn screening for a presymptomatic case in China: a case report

**DOI:** 10.1186/s13052-021-01136-y

**Published:** 2021-09-21

**Authors:** Yi Gan, Fei Yu, Haining Fang

**Affiliations:** 1 Pediatric Department, Maternal and Child Health Hospital of Hubei Province, Tongji Medical College, Huazhong University of Science and Technology, Wuhan, People’s Republic of China; 2 Neonatal Genetic Metabolic Disease Screening and Treatment Center in Hubei Province, Wuhan, People’s Republic of China

**Correction to: Ital J Pediatr 47, 154 (2021)**

**https://doi.org/10.1186/s13052-021-01094-5**

The original article [1] mistakenly omitted the labelling for Fig. 1I which has since been re-introduced.
